# Comparison of the MicroScan, VITEK 2, and Crystal GP with 16S rRNA sequencing and MicroSeq 500 v2.0 analysis for coagulase-negative Staphylococci

**DOI:** 10.1186/1471-2180-8-233

**Published:** 2008-12-23

**Authors:** Miyoung Kim, Se Ran Heo, Soon Hee Choi, Hyelin Kwon, Jeong Su Park, Moon-Woo Seong, Do-Hoon Lee, Kyoung Un Park, Junghan Song, Eui-Chong Kim

**Affiliations:** 1 Department of Laboratory Medicine, Seoul National University Hospital, 101 Daehang-no, Jongno-gu, Seoul, South Korea; 2 Department of Laboratory Medicine, Seoul National University Bundang Hospital, 300 Gumi-dong, Bundang-gu, Seongnam-si, Gyeonggi-do, South Korea; 3 Department of Laboratory Medicine, National Cancer Center, 809 Madu-1-dong, Ilsandong-gu, Goyang, Gyeonggi-do, South Korea; 4 Department of Laboratory Medicine, Seoul National University College of Medicine, 101 Daehang-no, Jongno-gu, Seoul, South Korea

## Abstract

**Background:**

Three phenotypic identification systems (MicroScan, VITEK 2, and Crystal GP) were evaluated for their accuracy to identify coagulase-negative staphylococci (CNS). A total of 120 clinical isolates confirmed to be CNS via 16S rRNA sequencing and analysis with the MicroSeq 500 v2.0 database were assessed.

**Results:**

The MicroScan, VITEK 2, and Crystal GP systems correctly identified 82.5%, 87.5%, and 67.5% of the isolates, respectively. Misidentification was the main problem in MicroScan (10.8%) and Crystal GP (23.3%) systems, whereas the main problem of VITEK 2 was low-level discrimination (7.5%).

**Conclusion:**

None of the 3 phenotypic systems tested could accurately and reliably identify CNS at the species level. Further verifications such as biochemical testing or 16S rRNA sequencing together with analysis using a comparable database might be helpful in this regard.

## Background

Because of their ubiquity and low virulence, coagulase-negative staphylococci (CNS) have generally been considered to be nonpathogens or simple contaminants. Recently, their clinical significance is being increasingly recognized with the elucidation of their pathogenicity. CNS can form biofilms and have been demonstrated to exhibit antibiotic resistance [1,2]. *S. epidermidis*, *S. haemolyticus*, and *S. lugdunensis*, to a lesser extent, are well-known etiological agents of implanted device-mediated infections [2-5]. *S. saprophyticus *can cause community-acquired infections of the uropoietic tract. Hence, species-level identification of CNS is necessary for correct guidance of clinicians in terms of appropriate treatment strategy, and a wide variety of identification methods have been proposed.

Many automated phenotypic identification systems are commercially available, including the MicroScan (Dade Behring, West Sacramento, CA, USA), VITEK 2 (bioMérieux, Maray l'Etoile, France), and Crystal GP (Becton Dickinson, Sparks, MD, USA) systems. On the basis of metabolic activities and/or morphological features, these systems enable microbiologists to identify bacterial isolates at the species level with greater ease, accuracy, and rapidity than that previously achieved [6]. However, these systems have several potential problems: (i) different strains in one species may not exhibit a specific characteristic, (ii) isolates from old cultures may not show the expected biochemical patterns, (iii) isolates from a host who has undergone long-term antimicrobial therapy may alter their typical biochemical characteristics, (iv) the same strain may not yield the same results in repeated tests, (v) the databases have data on a limited number of species, (vi) phenotypic variation may affect the accuracy of species-level identification by automated phenotypic systems, and (vii) phenotypic systems often suggest 2 or more designations with comparable probability levels [6-9].

Recently, genotypic methods are emerging as the new standard for bacterial identification in automated laboratories [4,6,7,10-14]. 16S ribosomal RNA (rRNA) sequences comprising both variable and conserved regions allow for clear differentiation between organisms not only at the species level but also at the subspecies level [10]. These sequences permit better identification of rare or phenotypically aberrant species as well as nonculturable bacteria. The MicroSeq 500 system (Applied Biosystems Inc. [ABI], Foster City, CA, USA) is a commercially available software system for 16S rRNA analysis.

Despite the increasing clinical significance of CNS and the growing use of automated phenotypic systems in clinical laboratories, few studies have systematically evaluated the accuracy of these systems [4,9,15]. In the present study, the accuracy of 3 commercial phenotypic systems (MicroScan, VITEK 2, and Crystal GP) for CNS identification was compared. 16S rRNA sequencing and MicroSeq 500 analysis were used as references [10-12,16-19].

## Results

### Clinical isolates

By using in-house primers and the MicroSeq 500 v2.0 database, we successfully identified all the 120 clinical isolates with more than 97% matches (data not shown). The identified species were as follows: 16 *S. capitis*, 4 *S. caprae*, 3 *S. cohnii*, 53 *S. epidermidis*, 4 *S. haemolyticus*, 25 *S. hominis*, 6 *S. lugdunensis*, 1 *S. saprophyticus*, 2 *S. simulans*, and 6 *S. warneri*. Blood culture specimens primarily contained *S. epidermidis*, whereas a variety of uncommon CNS were isolated from pitted keratolysis specimens. The result reflected the typical distribution of staphylococcal species detected routinely in microbiology laboratories.

### Identification using MicroScan

The overall performances of 3 phenotypic systems are summarized in Table 1 and the incorrect identification results are listed in Table 2. Of 120 isolates, correct identification, low-level discrimination and misidentification were 82.5% (99), 6.7% (8) and 10.8% (13), respectively. Nonidentification was not observed. Three *S. caprae *isolates were not correctly identified (isolate number: M211, M215, and M225), because the species *S. caprae *is not included in the MicroScan version 2.0 or 2.1 database. One of the *S. epidermidis *isolates (M106) was misidentified as *S. aureus*.

**Table 1:** Clinical isolates and identification results from MicroScan, VITEK2, and Crystal GP

Clinical Isolates (using MicroSeq 500)	No of isolates	Correct Identification^*a*^	Low-level Discrimination^*b*^	Misidentification^*c*^	Nonidentification^*d*^
		MicroScan			
		
*S. capitis*	16	13 (81.3%)	-	3 (18.8%)	-
*S. caprae*	4	-	-	4 (100.0%)	-
*S. cohnii*	3	1 (33.3%)	1 (33.3%)	1 (33.3%)	-
*S. epidermidis*	53	51 (96.2%)	-	2 (3.8%)	-
*S. haemolyticus*	4	3 (75.0%)	1 (25.0%)	-	-
*S. hominis*	25	23 (92.0%)	2 (8.0%)	-	-
*S. lugdunensis*	6	2 (33.3%)	1 (16.7%)	3 (50.0%)	-
*S. saprophyticus*	1	1 (100.0%)	-	-	-
*S. simulans*	2	2 (100.0%)	-	-	-
*S. warneri*	6	3 (50.0%)	3 (50.0%)	-	-
Subtotal	120	99 (82.5%)	8 (6.7%)	13 (10.8%)	-
		
		VITEK 2			
		
*S. capitis*	16	16 (100.0%)	-	-	-
*S. caprae*	4	3 (75.0%)	-	1 (25.0%)	-
*S. cohnii*	3	3 (100.0%)	-	-	-
*S. epidermidis*	53	47 (88.7%)	6 (11.3%)	-	-
*S. haemolyticus*	4	4 (100.0%)	-	-	-
*S. hominis*	25	22 (88.0%)	1 (4.0%)	2 (8.0%)	-
*S. lugdunensis*	6	5 (83.3%)	1 (16.7%)	-	-
*S. saprophyticus*	1	1 (100.0%)	-	-	-
*S. simulans*	2	1 (50.0%)	1 (50.0%)	-	-
*S. warneri*	6	3 (50.0%)	-	3 (50.0%)	-
Subtotal	120	105 (87.5%)	9 (7.5%)	6 (5.0%)	-
		
		Crystal GP			
		
*S. capitis*	16	13 (81.3%)	-	3 (18.8%)	-
*S. caprae*	4	-	-	4 (100.0%)	-
*S. cohnii*	3	3 (100.0%)	-	-	-
*S. epidermidis*	53	49 (92.5%)	-	3 (5.7%)	1 (1.9%)
*S. haemolyticus*	4	4 (100.0%)	-	-	-
*S. hominis*	25	1 (4.0%)	-	15 (60.0%)	9 (36.0%)
*S. lugdunensis*	6	2 (33.3%)	-	3 (50.0%)	1 (16.7%)
*S. saprophyticus*	1	1 (100.0%)	-	-	-
*S. simulans*	2	2 (100.0%)	-	-	-
*S. warneri*	6	6 (100.0%)	-	-	-
Subtotal	120	81 (67.5%)	-	28 (23.3%)	11 (9.2%)

Correct identification^*a*^: single, unambiguous, correct identification at the species level

Low-level discrimination^*b*^: two or more possible species level identification including the correct one

Misidentification^*c*^: genus or species-level identification different from that obtained with the reference method

Nonidentification^*d*^: no identification or unidentification

**Table 2:** Identification results with percent probability or confidence factor of single wrong identification or multiple identifications in three phenotypic systems

	MicroSeq 500	MicroScan	VITEK2	Cystal GP
M102	*S. epidermidis*	*S. epidermidis *91.36%	*S. epidermidis *97.10%	*S. intermedius*
M104	*S. epidermidis*	*S. xylosus *95.12%	*S. epidermidis *99.00%	*S. epidermidis*
M106	*S. epidermidis*	*S. aureus *87.13%	*S. epidermidis *99.00%	*S. epidermidis*
M107	*S. capitis*	*S. simulans *88.95%	*S. capitis *98.78%	*S. capitis*
M112	*S. epidermidis*	*S. epidermidis *99.99%	*S. epidermidis *99.00%	*K. sedentarius*
M117	*S. lugdunensis*	*S. haemolyticus *90.39%	*S. lugdunensis *99.00%	Nonidentification
M118	*S. lugdunensis*	*S. haemolyticus *96.86%	*S. lugdunensis *90.37%	*S. haemolyticus*
M120	*S. hominis*	*S. hominis *94.57%	*S. hominis *95.00%	Nonidentification
M123	*S. epidermidis*	*S. epidermidis *98.20%	*S. hominis*, *S. epidermidis*, *A. viridans *33.82%, 33.09%, 33.09%	*S. epidermidis*
M124	*S. epidermidis*	*S. epidermidis *99.25%	*S. hominis*, *S. epidermidis *50.27%, 49.73%	Nonidentification
M125	*S. epidermidis*	*S. epidermidis *98.20%	*S. hominis*, *S. epidermidis *50.27%, 49.73%	S. epidermidis
M126	*S. epidermidis*	*S. epidermidis *99.99%	*S. hominis*, *S. epidermidis*, *A. viridans *33.71%, 33.33%, 32.96%	*S. epidermidis*
M127	*S. epidermidis*	*S. epidermidis *99.99%	*S. hominis*, *S. epidermidis *50.28%, 47.92%	*S. epidermidis*
M128	*S. hominis*	*S. hominis *94.58%	*S. hominis *94.86%	*S. saprophyticus*
M130	*S. capitis*	*S. epidermidis *90.20%	*S. capitis *92.00%	*S. capitis*
M137	*S. epidermidis*	*S. epidermidis *95.28%	*S. hominis*, *S. epidermidis *50.56%, 49.44%	*S. epidermidis*
M138	*S. capitis*	*S. capitis *95.28%	*S. capitis *98.86%	*S. auricularis*
M139	*S. capitis*	*S. capitis *99.60%	*S. capitis *98.86%	*S. hominis*
M141	*S. epidermidis*	*S. epidermidis *99.99%	*S. epidermidis *98.16%	*S. hominis*
M149	*S. capitis*	*S. epidermidis *90.20%	*S. capitis *92.00%	*S. capitis*
M153	*S. hominis*	*S. hominis *97.44%	*S. hominis *99.00%	Nonidentification
M154	*S. hominis*	*S. hominis *94.62%	*S. hominis *98.35%	Nonidentification
M155	*S. hominis*	*S. hominis *97.44%	*S. hominis *97.21%	*S. warneri, S. saprophyticus *0.5574, 0.4415
M156	*S. hominis*	*S. hominis *95.00%	*S. hominis *97.00%	*S. saprophyticus*
M157	*S. warneri*	*S. warneri*, *S. cohnii*, *S. capitis*, etc. 60.92%, 15.78%, 12.67%.	*S. cohnii*, *S. vitulinus *50.54%, 49.46%	*S. warneri*
M158	*S. warneri*	*S. warneri *90.92%	*S. cohnii *90.54%	*S. warneri*
M160	*S. hominis*	*S. hominis*, *S. capitis*, *S. warneri*, etc. 37.58%, 24.33%, 20.21%.	*S. hominis *93.79%	Nonidentification
M161	*S. hominis*	*S. hominis *94.57%	*S. hominis *96.98%	*S. saprophyticus*
M162	*S. caprae*	*S. hominis *92.35%	*S. auricularis *90.40%	*S. saprophyticus*
M163	*S. hominis*	*S. hominis *98.82%	*S. hominis *98.50%	Nonidentification
M166	*S. hominis*	*S. hominis *85.00%	*S. hominis *98.82%	Nonidentification
M167	*S. hominis*	*S. hominis *93.05%	*S. caprae *96.97%	*S. caprae*
M176	*S. simulans*	*S. simulans *96.77%	*S. simulans*, *S. warneri *50.28%, 49.72%	*S. simulans*
M17	*S. hominis*	*S. simulans *99.09%	*S. haemolyticus *96.74%	*S. haemolyticus*
M179	*S. hominis*	*S. epidermidis*, *S. hominis *68.34%, 31.66%	*S. hominis *94.88%	Nonidentification
M180	*S. hominis*	*S. hominis *92.77%	*S. hominis *93.56%	*S. warneri*
M181	*S. hominis*	*S. hominis *92.77%	*S. hominis*, *S. warneri *50.27%, 49.73%	*S. warneri*
M182	*S. hominis*	*S. hominis *94.57%	*S. hominis *95.00%	Nonidentification
M183	*S. hominis*	*S. hominis *88.68%	*S. hominis *91.00%	*S. saprophyticus*
M184	*S. hominis*	*S. hominis *92.13%	*S. hominis *99.00%	*S. intermedius*
M185	*S. hominis*	*S. hominis *96.01%	*S. hominis *96.81%	*S. capitis*
M186	*S. hominis*	*S. hominis *98.50%	*S. hominis *95.00%	*S. aureus, S. intermedius*
M187	*S. hominis*	*S. hominis *98.50%	*S. hominis *99.00%	Nonidentification
M203	*S. hominis*	*S. hominis *97.44%	*S. hominis *97.27%	*S. cohnii*
M205	*S. warneri*	*S. warneri*, *S. capitis*, *S. hominis *86.37%, 7.96%, 5.67%	*S. saphrophyticus *98.03%	*S. warneri*
M207	*S. lugdunensis*	*S. haemolyticus *98.08%	*S. lugdunensis *99.00%	*S. lugdunensis*
M208	*S. haemolyticus*	*S. haemolyticus*, *S. simulans*, *S. warneri *54.38%, 41.97%, 3.66%	*S. haemolyticus *94.74%	*S. haemolyticus*
M209	*S. hominis*	*S. hominis *97.44%	*S. hominis *97.76%	*S. cohnii*
M210	*S. cohnii*	*S. cohnii*, *S. xylosus *67.28%, 29.12%	*S. cohnii *99.00%	*S. cohnii*
M211	*S. caprae*	*S. aureus*, *S. capitis *69.87%, 29.25%	*S. caprae *98.60%	*S. intermidius*
M212	*S. capitis*	*S. capitis *99.11%	*S. capitis *98.56%	*S. vitulus*
M213	*S. warneri*	*S. auricularis*, *S. hominis*, *S. warneri *45.13%, 27.55%, 24.55%	*S. warneri *95.00%	*S. warneri*
M214	*S. lugdunensis*	*S. lugdunensis *98.81%	*S. lugdunensis *95.00%	*S. simulans*
M215	*S. caprae*	*S. capitis*, *S. aureus *62.82%, 35.76%	*S. caprae *99.00%	*S. cohnii*
M216	*S. hominis*	*S. hominis *97.44%	*S. hominis *97.76%	*M. kristinae*
M221	*S. lugdunensis*	*S. lugdunensis*, *S. haemoiyticus*, *S. hominis *62.62%, 28.34%, 9.04%	*S. lugdunensis*, *S. warneri *50.00%, 50.00%	*S. lugdunensis*
M222	*S. lugdunensis*	*S. lugdunensis *96.30%	*S. lugdunensis *91.26%	*S. simulans*
M224	*S. cohnii*	*S. xylosus *91.70%	*S. cohnii *99.00%	*S. cohnii*
M225	*S. caprae*	*S. warneri*, *S. simulans*, *S. epidermidis *70.19%, 13.84%, 12.32%	*S. caprae *99.00%	*S. capitis*

### Identification using VITEK 2

In the VITEK 2 analysis, correct identification, low-level discrimination and misidentification were 87.5% (105), 7.5% (9) and 5.0% (6), respectively. Nonidentification did not occur. Of note, the analysis of 6 *S. epidermidis *isolates (6/53, 11.3%) resulted in low-level discriminations – multiple identifications as *S. hominis*, *S. epidermidis*, or *Aerococcus viridans *with low percent probabilities, suggesting *S. hominis *with the highest percent probability followed by *S. epidermidis *(M123, M124, M125, M126, M127, M137).

### Identification using Crystal GP

Crystal GP is not as fully automated as the other two, as the reading of the biochemical reaction depends on manual reading. In order to prevent the bias resulting from manual reading, the biochemical reaction results were read by two different technicians, followed by repeated tests on wrongly identified isolates or those with multiple identifications or nonidentifications. The identification results of 81 isolates (67.5%) using the Crystal GP system agreed with those of the MicroSeq 500 system with confidence factors of more than 0.9 (correct identifications). In total, 23.3% (28) were misidentified, and 9.2% (11) could not be identified (nonidentification). Low-level discrimination was not observed. Misidentifications at the genus level occurred for 1 *S. epidermidis *and 1 *S. hominis *isolate (M112, M216). The Crystal GP system correctly identified all the isolates of *S. cohnii*, *S. haemolyticus*, *S. saprophyticus*, *S. simulans*, and *S. warneri*.

## Discussion

With the increased attention being given to the clinical significance of CNS, clinical laboratories must correctly identify CNS at the species level by using reliable and reproducible methods. Because clinical microbiology laboratories are increasingly becoming dependent on automated systems, the accuracy of these systems must be investigated. In the present study, the accuracy of 3 phenotypic identification systems for CNS identification was tested and compared with the sequencing of 16S rRNA.

MicroScan and VITEK 2 showed similar concordance rate to MicroSeq 500. VITEK 2 showed a slightly higher "correct identification" rate (82.5% vs. 87.5%, respectively). When the low-level discrimination results that included the correct identification were considered together, the concordance rates were 89.2% (107/120) for MicroScan and 95.0% (114/120) for VITEK 2. Crystal GP had the lowest correct identification rate (67.5%). All 3 systems had misidentifications: single but incorrect identification results with higher than acceptable probability of over 85%, which is consistent with the system manufacturer's reported homology value without additional verification, or with higher than a confidence factor of 0.9. This indicates that CNS identifications made using these phenotypic systems cannot always be considered accurate, regardless of the reported probability.

In MicroScan analysis, none of the *S. caprae *identifications were correct (3 low-level identifications and 1 misidentification). This is because the species *S. caprae *is not included in the MicroScan database, which illustrates the inappropriateness of this database. Ambiguous identifications, including low-level discriminations and misidentifications, were observed for 8 out of 10 species (with the exception of *S. saprophyticus *and *S. warneri*) with the MicroScan system, compared with 6 ambiguous identifications with the VITEK2 system and 5 with the Crystal GP system. This finding was consistent with results reported by other researchers who found that the MicroScan system has greater accuracy in the identification of *S. epidermidis *and *S. saprophyticus *than for identification of other species [20-22]. The other notable finding of the present study is that 1 *S. epidermidis *isolate from blood culture was misidentified as *S. aureus *with 87.5% probability, which could have delayed appropriate treatment for the patient.

In VITEK 2 analysis, low-level discriminations of 6 *S. epidermidis *isolates (11.3% of *S. epidermidis *isolates) occurred, compared to higher correct identification rates with the MicroScan and Crystal GP systems (96.2% and 92.5%, respectively). Some researchers reported different sort of difficulties in identifying *S. epidermidis *with VITEK 2 [9,23,24]. However, others reported high accuracy of VITEK 2 for the identification of staphylocccal species [25,26]. The performance of VITEK 2 in the identification of CNS including *S. epidermidis *seems not to be determined yet, and the additional testing such as manual biochemical testing or sequencing is required for the accurate diagnosis.

The Crystal GP system provides no guidelines for the interpretation of the confidence factor, therefore, all isolates with confidence factors lower than 0.9 were retested. In spite of repeated testing, this system had the lowest correct identification rate and the highest rate of nonidentification.

One limitation of this study is that additional biochemical testing was not preformed. 16S rRNA sequencing might not be a perfect method for interspecies discrimination. However, not all the suggested biochemical tests are available in many automated laboratories or based on our experience, manual testing could not resolve the ambiguity of CNS identification in many cases. To improve reliability in this study, all isolates with discrepancies or uncertain results were retested using isolates preserved in skim milk at -70°C.

## Conclusion

None of the tested systems (MicroScan, VITEK 2, Crystal GP) are able to accurately identify all of the staphylococcal species evaluated in this study. Additional verification via molecular testing or suggested biochemical tests may prove helpful for correct CNS identification.

## Methods

### Clinical isolates

A total of 120 clinical isolates (95 blood culture isolates, 25 skin isolates from pitted keratolysis patients) were used in this study [27]. To exclude simple contaminants, only isolates of blood cultures that grew in more than 2 of 3 blood cultures were included. The pitted keratolysis specimen was obtained after repeated washing of the lesion with normal saline by scratching the lesion with a sterile blade into a sterile conical tube. The isolates were tested within 24 hours. For additional tests or repeated tests, the isolates were suspended in skim milk, stored at -70°C and retested. All of the isolates were confirmed to be CNS by use of 16S rRNA sequencing with MicroSeq 500 software (version 2.0) with over 97% matches.

### Phenotypic identification of CNS

Three phenotypic systems were evaluated: MicroScan Pos Combo panel type 1A (Dade Behring, West Sacramento, CA, USA), VITEK 2 GP-ID cards (bioMérieux, Marcy l'Etoile, France), and Crystal Gram Positive ID systems (Becton Dickinson, Sparks, MD, USA). Bacterial suspensions were prepared from well-isolated colonies. The colonies were suspended in the broth provided by each company, and the turbidity was adjusted to 0.5 McFarland. MicroScan Pos Combo panel type 1A was inoculated according to the manufacturer's recommendations and processed with a MicroScan Walkaway 96 apparatus. The blood culture isolates were analyzed using a MicroScan LabPro system (version 2.0) and the skin isolates were analyzed with version 2.01. VITEK 2 GP-ID cards were used in combination with a VITEK 2 system (version 4.02) for all isolates. The Crystal GP results were read manually by 2 different technicians using the Crystal GP Identification Color Chart.

The MicroScan system classifies identification results into 4 categories by percent probability: species identified with high probability (> 85%), species identified with low probability (< 85%), very rare biotype, and group ID (> 85% but genus or group identified only). The VITEK 2 system categorizes the identification results by probability: excellent (96%–99%), very good (93%–95%), good (89%–92%), acceptable (85%–88%), low discrimination (2–3 identifications with 100% probability in total), and nonidentification. The Crystal GP system gives confidence factors instead of probability; however, there are no such criteria in terms of the confidence factor. When the confidence factor is low, Crystal GP reports non-identification, which necessitates additional manual testing.

The results of incorrect identifications (not concordant to that of MicroSeq 500) regardless of probability, identification with unacceptable probability (lower than 85% for MicroScan and VITEK 2) or low confidence factor (lower than 0.9 for Crystal GP), or nonidentification were retested with colonies that were stored in skim milk at -70°C. Those with incorrect, or consistently unacceptable or nonidentifiable results are listed in Table 2. The confidence factor of the Crystal GP system is not included, as there are no guidelines for these interpretations.

### 16S rRNA sequencing

To extract bacterial DNA, InstaGene Matrix (Bio-Rad Laboratories, Hercules, CA, USA) was used. Two or 3 colonies of each isolate were suspended in 100 μl sterile distilled water and centrifuged for 5 min at 15,000 rpm. A 200 μl aliquot of InstaGene Matrix (Bio-Rad Laboratories, Hercules, CA, USA) was added to the sediment. The mixture was heat-lysed for 5 min at 100°C, cooled at room temperature, and centrifuged for 3 min at 15,000 rpm. The supernatant (2.5 μl) of each bacterial extract was used for successive amplification procedures. Polymerase chain reaction (PCR) was conducted in a total volume of 25 μl containing 2.5 mM dNTP, 10 pmol of each PCR primer, 0.6 units Taq polymerase, 2.5 μl 10 × PCR buffer with 15 mM MgCl2 (Takara Bio, Inc., Shiga, Japan), and 2.5 μl template. In-house primers were designed using the LightCycler Probe Design software (version 2.0) (Roche, Penzberg, Germany), using published studies as a reference [8,9]. The primers used for amplification were MSQ-F (5'-TGA AGA GTT TGA TCA TGG CTC AG-3') and MSQ-R (5'-ACC GCG GCT GCT GGC AC-3'). The PCR conditions were as follows: 10 min of initial denaturation at 95°C, followed by 35 cycles of annealing of 30s at 95°C, 30s at 60°C, 45s at 72°C, and a final 10-minute extension at 72°C. Gel electrophoresis was used to detect positive PCR signals and to confirm the length of the amplicon. Prior to sequencing, the PCR products were purified using ExoSAP-IT reagent (USB Corporation, Cleveland, Ohio, USA) according to the manufacturer's instructions. Forward and reverse sequencing reactions were conducted for each of the amplified products. The sequencing reaction consisted of 10 μl MicroSeq 500 sequencing mix (containing 1.6 pmol of MSQ-F and MSQ-R primers), 2.9 μl sterile distilled water, and 1 μl purified PCR product. Sequencing reactions were performed using Big Dye terminator reagents on an ABI Prism 3130xl Genetic Analyzer (Applied Biosystems Inc., Foster City, CA, USA) according to standard automated sequencer protocols.

### Sequence analysis

MicroSeq 500 version 2.0 software was used for sequence analysis. The analysis steps were as follows: (i) the forward and reverse sequences were assembled into one consensus sequence, (ii) the consensus sequence was edited to resolve discrepancies between the 2 strands by evaluating the electropherograms, and (iii) the consensus sequence was compared with sequences in the MicroSeq 500 database. The comparison, using the full alignment tool of the MicroSeq 500 software, generated a list of the closest matches with a distance score, which indicates the percentage difference between the unknown sequence and the database sequence. Only when the consensus sequence had more than a 97% match with that of the MicroSeq 500 results, the identification results were considered to be acceptable [7,10].

### Analysis of the results

The results of 16S rRNA sequencing and MicroSeq 500 analysis were regarded as the definitive identification [10-12,16-19]. The species-level identification and percent probability from phenotypic systems were all taken into consideration. The concordance of the results of the phenotypic systems with those of MicroSeq 500 were classified into 4 categories according to the following definitions: (i) correct identification (unambiguous, single identification identical to that of MicroSeq 500 at the species level, with > 85% probability in MicroScan and VITEK 2 or 0.9 confidence factor); (ii) low-level discrimination (2 or more possible species-level identifications, including the correct one, with probability < 85% in MicroScan and VITEK 2 or the low confidence factor under 0.9 in Crystal GP); (iii) misidentification (genus-level or species-level identification different from that obtained with the reference method) and (iv) nonidentification (isolates that were unable to be identified at the species level in spite of the repeated testing).

## Authors' contributions

MK designed the study, helped with the phenotypic identification and molecular studies, and wrote the manuscript. SHC performed sample collection and phenotypic identification. SRH, HK and JSP carried out the molecular genetic studies, participated in the sequence alignment. MWS, DHL, JS and ECK helped design the study and helped with technical issues. KUP conceived of study, and participated in its design and coordination, helped to draft the manuscript and reviewed the manuscript. All of the authors have read and approved the final manuscript.
